# Myeloid Ezh2 Deficiency Limits Atherosclerosis Development

**DOI:** 10.3389/fimmu.2020.594603

**Published:** 2021-01-26

**Authors:** Annette E. Neele, Hung-Jen Chen, Marion J. J. Gijbels, Saskia van der Velden, Marten A. Hoeksema, Marieke C. S. Boshuizen, Jan Van den Bossche, Anton T. Tool, Hanke L. Matlung, Timo K. van den Berg, Esther Lutgens, Menno P. J. de Winther

**Affiliations:** ^1^ Department of Medical Biochemistry, Amsterdam Cardiovascular Sciences, Amsterdam Infection and Immunity, Amsterdam UMC, University of Amsterdam, Amsterdam, Netherlands; ^2^Department of Pathology and Department of Molecular Genetics, CARIM, Maastricht University, Maastricht, Netherlands; ^3^Department of Cellular and Molecular Medicine, School of Medicine, University of California San Diego, La Jolla, CA, United States; ^4^Department of Molecular Cell Biology and Immunology, Amsterdam Cardiovascular Sciences, Amsterdam Gastroenterology Endocrinology Metabolism, Amsterdam UMC, Vrije Universiteit Amsterdam, Amsterdam, Netherlands; ^5^Department of Blood Cell Research, Sanquin Research and Landsteiner Laboratory, Amsterdam UMC, University of Amsterdam, Amsterdam, Netherlands; ^6^Institue for Cardivascular Prevention (IPEK), Ludwig Maximilias University (LMU), Munich, Germany

**Keywords:** atherosclerosis, epigenetic, histone modification, H3K27, macrophage, polycomb, PRC2

## Abstract

Macrophages define a key component of immune cells present in atherosclerotic lesions and are central regulators of the disease. Since epigenetic processes are important in controlling macrophage function, interfering with epigenetic pathways in macrophages might be a novel approach to combat atherosclerosis. Histone H3K27 trimethylation is a repressive histone mark catalyzed by polycomb repressive complex with EZH2 as the catalytic subunit. EZH2 is described to increase macrophage inflammatory responses by supressing the suppressor of cytokine signaling, *Socs3*. We previously showed that myeloid deletion of *Kdm6b*, an enzymes that in contrast to EZH2 removes repressive histone H3K27me3 marks, results in advanced atherosclerosis. Because of its opposing function and importance of EZH2 in macrophage inflammatory responses, we here studied the role of myeloid EZH2 in atherosclerosis. A myeloid-specific *Ezh2* deficient mouse strain (*Ezh2*^del^) was generated (LysM-cre+ x *Ezh2*^fl/fl^) and bone marrow from *Ezh2*^del^ or *Ezh2*^wt^ mice was transplanted to *Ldlr*^-/-^ mice which were fed a high fat diet for 9 weeks to study atherosclerosis. Atherosclerotic lesion size was significantly decreased in *Ezh2*^del^ transplanted mice compared to control. The percentage of macrophages in the atherosclerotic lesion was similar, however neutrophil numbers were lower in *Ezh2*^del^ transplanted mice. Correspondingly, the migratory capacity of neutrophils was decreased in *Ezh2*^del^ mice. Moreover, peritoneal *Ezh2*^del^ foam cells showed a reduction in the inflammatory response with reduced production of nitric oxide, IL-6 and IL-12. In Conclusion, myeloid *Ezh2* deficiency impairs neutrophil migration and reduces macrophage foam cell inflammatory responses, both contributing to reduced atherosclerosis.

## Introduction

Atherosclerosis is a chronic lipid-driven inflammatory disorder of the arteries. Macrophages define a key component of immune cells present in atherosclerotic lesions and are important regulators of disease development and outcome (1). Unraveling pathways that are involved in the control of macrophage inflammatory responses in atherosclerosis will lead to a better understanding of disease. Since epigenetic processes are important modulators of macrophage function, we postulate that interference with epigenetic enzymes in macrophages might be a novel approach to modify macrophage function and influence atherosclerosis development (2, 3).

Histone H3K27 trimethylation (H3K27me3) is a repressive histone mark generated by the polycomb repressive complex 2 (PRC2), which core consists of Embryonic ectoderm development (EED), SUZ12, RpAp46/48 and Enhancer of the zeste homolog 1 (EZH1) or EZH2 (4). EZH1/2 contain a SET domain, which is necessary for the methyltransferase activity of the PRC2 complex (5, 6). Both EZH1 and EZH2 homologs can form similar PRC2 complexes but their differential function remains unclear. PRC2-EZH2 catalyzes mainly H3K27me2/3 methylation and *Ezh2* knockdown affects global H3K27me3 levels, while EZH1 in the PRC2 complex performs this function weakly (7). EZH2 has been studied mainly in relation to developmental biology and cancer research. In addition, EZH2 controls stem cell proliferation and differentiation and it was demonstrated that EZH2 regulates T cell differentiation and plasticity (8).

EZH2 is a strong regulator macrophage inflammatory responses (9, 10). Macrophage *Ezh2* deficiency was shown to inhibit pro-inflammatory gene expression *via* upregulation of suppressor of cytokine signaling 3 (*SOCS3*). Consequently, mice lacking *Ezh2* in their macrophages showed improved outcome in models for autoimmune inflammation, experimental autoimmune encephalomyelitis (EAE) and colitis. The H3K27 demethylase KDM6b (also known as Jmjd3) removes repressive histone marks and is also an important regulator of macrophage activation (11–17). We previously showed that myeloid *Kdm6b* deficiency in mice results in advanced atherosclerosis (18). Since the H3K27 methyltransferases have opposing effects on this histone mark and the fact that EZH2 controls macrophage inflammatory responses, we hypothesized that inhibition of the H3K27 methyltransferase EZH2 in myeloid cells improves atherosclerosis outcome. To test this, we generated myeloid specific *Ezh2* knockout mice and studied atherosclerosis progression. We found that atherosclerosis was significantly reduced in *Ezh2*-deficient mice, correlating with impaired neutrophil migration and reduced foam cell inflammatory responses.

## Materials and Methods

### Atherosclerosis Experiment

Low-density lipoprotein receptor knock out mice (*Ldlr*^-/-^) (C57BL/6 background, Jackson laboratories) were used to study atherosclerosis, since these mice are prone to develop atherosclerosis in the presence of a high fat diet (HFD). A bone marrow transplantation (BMT) was performed with either LysM-cre^+^ *Ezh2*^fl/fl^ mice (*Ezh2^d^*^el^) or LysM-cre^-^ *Ezh2*^fl/fl^ littermates (*Ezh2*^wt^). *Ezh2*^fl/fl^ mice were described before and crossbreeding with LysM-cre was performed in our mice facility (19). Briefly, 40 (20 per group), 10-week-old female *Ldlr*^-/-^ mice were randomly divided over filter-top cages and provided with antibiotics water [autoclaved tap water with neomycin (100 mg/l, Sigma) and polymyxin B sulfate (60,000 U/l, Invitrogen)] from 1 week pre-BMT till 5 weeks post-BMT. The animals received 2 × 6 Gy total body irradiation on two consecutive days. Bone marrow was isolated from 4 *Ezh2*^del^ and 4 *Ezh2*^wt^ littermates, resuspended in RPMI1640 (Gibco) with 5 U/ml heparin and 2% iFCS (Gibco), and 5×10^6^ bone marrow cells were injected intravenously per irradiated mouse. Five weeks after the BMT, the mice were put on a HFD (0.15% cholesterol, 16% fat, Arie Blok Diets) for 9 weeks. After sacrifice, hearts were isolated and frozen in Tissue-Tek (Sakura) for histology. Blood samples were taken before the start of the diet and four days before sacrifice (week 8) for lipid profiling and immune cell flow cytometry. Blood was withdrawn from mice which were fasted for 3 h. Bone marrow transplantation efficiency was determined with quantitative PCR for the *Ldlr* on DNA isolated from blood (GE Healthcare). Four mice were excluded from the analysis due to inefficient bone marrow transplantation (≤80%). Three mice were euthanized before the end of experiment since they reached their humane endpoints as a result of excessive weight loss (>15% weight loss since starting weight). Two additional mice were excluded from the analysis due to insufficient tissue quality and one mouse was excluded after outlier detection. A final of 15 mice per group was used for the statistical analysis. All animal experiments were conducted at the University of Amsterdam and approved (permit: DBC10AH) by the Committee for Animal Welfare of the Amsterdam UMC, location Academic Medical Center, University of Amsterdam.

### Histochemistry

Atherosclerotic lesions from the heart were cut in sections of 7 μm on a Leica 3050 cryostat at −25°C. Cross sections of every 42 μm were stained with Toluidin Blue (0.2% in PBS, Sigma-Aldrich) to determine lesion size. Lesion size was measured by use of adobe photoshop CS4 and the sum of the three valves is presented. Sirius red staining was performed for 30 min to detect collagen (0.05% direct Red in saturated picric acid, Sigma). Collagen content was quantified as the percentage collagen of total lesion size. For immunohistochemistry, slides were fixed in acetone and blocked with Avidin/Biotin Blocking Kit (Vector Laboratories). Hereafter, cells were incubated with MOMA-2 (1:4,000, AbD Serotec) to stain macrophages. Biotin-labeled rabbit anti–rat antibody (1:300, Dako) was used as a secondary antibody. The signal was amplified using ABC kit (Vector Laboratories) and visualized with the AEC kit (Vector Laboratories). Macrophages were quantified as the percentage of total lesion size. Neutrophils were stained with Ly6G antibody (BD biosciences) and incubated overnight at 4°C. Fluorescent rabbit anti-rat antibody was used as a secondary antibody (1:500, Thermo Fisher Scientific) and incubated for 1 h at room temperature (RT). Nuclei were stained with DAPI (1:5,000, Thermo Fisher Scientific) and incubated for 15 min at RT. Slides were mounted and fluorescence was measured using a Leica DM300 microscope. Neutrophil numbers were counted manually and corrected for lesion size. Necrosis area was measured based on Toluidine Blue staining by our pathologist and corrected for total lesion area.

### Flow Cytomery

150 μl of blood was withdrawn from mice *via* tail vein incision before the start of the diet and four days prior to sacrifice and added to 20 μl of 0.5 M EDTA (Sigma-Aldrich). Blood was withdrawn from mice which were fasted for 3 h. The blood was centrifuged (10 min, 4°C, 2,000 rpm) to separate the plasma from blood cells and plasma cholesterol and triglyceride levels were enzymatically measured according to the manufacturer’s protocol (Roche). Blood was further used for flow cytometry to assess relative leukocyte counts. Red blood cells were lysed by adding 5 ml of erythrocyte lysis buffer (8.4 g of NH_4_Cl, 0.84 g of NaHCO_3_, and 0.37 g of EDTA in 1 L MilliQ) for 15 min at RT. PBS was added to stop the reaction and cells were centrifuged. This was repeated until the pellet was not red anymore. White blood cells were used for flow cytometry. First Fc receptors were blocked with CD16/CD32 blocking antibody (1:100, eBioscience) in FACS buffer (0.5% BSA, 2mM EDTA in PBS). Hereafter cells were incubated with the appropriate antibodies for 30 min at RT (**Supplemental Table 1**). Cells were washed once and resuspended in FACS buffer. Fluorescence was measured with a BD Canto II and analyzed with FlowJo software. Immune cells were gated based on CD45^+^ expression and the following cell types were distinguished: Monocytes (CD11b^+^ and Ly6G^-^), Neutrophils (CD11b^+^ and Ly6G^+^), B cells (CD19^+^), and T cells (CD3^+^).

### Bone Marrow-Derived Macrophage Culture

Bone marrow was isolated from femurs and tibia of *Ezh2*^wt^ or *Ezh2*^del^ mice by flushing. The cells were cultured in RPMI-1640 with 25 mM HEPES and 2 mM L-glutamine (Life Technologies) which was supplemented with 10% FCS, penicillin (100 U/ml), streptomycin (100 µg/ml) (all Gibco) and 15% L929-conditioned medium. Cells were cultured for 8 days to generate bone marrow-derived macrophages (BMDMs) on bacteriologic plastic plates. On day 8, macrophages were resuspended at a density of 10^6^ cells/ml and plated in suspension culture plates allowing adherence for 6 h (Greiner). Next, cells were stimulated with LPS (10 ng/ml) for 6 h or left unstimulated where after the supernatant was collected for neutrophil migration experiments. Cultured BMDMs were also used for qPCR analysis, western blots and the H3K27 histone methyltransferase activity assay.

### RNA Isolation and Quantitive PCR Analysis

RNA from BMDMs or PEMs was isolated with the High Pure RNA isolation kits (Roche) from 500,000 cells. 400 ng of RNA was used for cDNA synthesis with iScript (BioRad). qPCR was performed with 4 ng of cDNA using Sybr Green Fast on a ViiA7 PCR machine (Applied Biosystems). *Ezh1*, *Ezh2*, *Cxcl1*, and *Cxcl2* gene expression was normalized to the mean of the two housekeeping genes *Ppia* and *Rplp0*. Primer sequences are available on request.

### Western Blot Analysis

NP40 lysis buffer (Invitrogen) was used for whole cell lysates, supplemented with protease inhibitor cocktail (1:25; Roche). Cells were lysed for 30 min on ice. Hereafter, cells were spun down for 10 min, 4°C at maximum speed and the supernatant was collected and used for western blot analysis for EZH2 protein expression. Histone extractions were used for H3K27Me3 Western blotting. For the histone extractions, cells were resuspended in Triton Extraction Buffer [PBS containing 0.5% Triton X 100 (v/v) and 0.02% (w/v) NaN_3_ supplemented with protease inhibitor cocktail (1:25; Roche)] and lysed on ice for 10 min. Cells were spun down for 10 min at 4°C at 2000 rpm. The supernatant was discarded and the pellet was resuspended in 0.2N HCl and histones were extracted overnight at 4°C on a rotator. Samples were centrifuged for 10 min, 4°C at 2000 rpm and the supernatant was used. Whole cell lysates were diluted with 6x reducing sample buffer (374 mM Tris, 6% SDS, 0.05% Bromophenol Blue, 20% Glycerol, 10% β-mercaptoethanol) and histone extracts with 2x reducing sample buffer (4% SDS, 20% glycerol, 0.125 M Tris-HCl, 10% β-mercaptoethanol, and 0.004% Serva Blue, pH 6.8) and boiled for 10 min at 95°C. Hereafter samples were loaded on a NuPAGE^®^ Novex 4-12% Bis-Tris protein gel and ran for 1.5 h in MOPS buffer for EZH2 western blotting or MES buffer for H3K27Me3 western blotting (NuPAGE^®^ MOPS or MES SDS Running Buffer 20x (Invitrogen) in demi water) at 100 V. The gel was transferred to a nitrocellulose membrane (Bio-Rad) and blotted for 2 h at 30 V in transfer buffer (20x transferbuffer NuPAGE^®^ (Invitrogen), 20% Methanol in demi water). The membrane was blocked in 5% milk (Milk powder (Elk) in TBS-T) for 1 h and hereafter the blot for EZH2 was cut and incubated with the primary antibodies overnight in 1% milk at 4°C. The primary antibodies we used were anti-EZH2 (1:1,000; Bioke) and anti-α-tubulin (1:5,000; Sigma) and for histone extracts anti-H3K27Me3 (1:1,000; bioconnect) and anti-H3 (1:5,000; Cell Signaling Technology)The next day, blots were washed and incubated for 1 h at RT with the appropriate HRP-conjugated secondary antibody in 1% milk (1:5000 Dako). Blots were visualized using ECL substrate kit (Thermo Scientific).

### H3K27 Methyltransferase Activity Assay

The H3K27 histone methyltransferase activity assay was performed with the EpiQuikTM assay kit on nuclear lysates following manufactures instructions (Epigentek). Absorbace was read on a microplate reader at 450 nm. HMT activity was calculated by the following formula: Activity (OD/h/mg)=(OD(sample-blank)/protein amount (µg) x incubation time substrate (h) × 1,000.

### Neutrophil Migration

Neutrophils were isolated from female mouse bone marrow of *Ezh2*^wt^ and *Ezh2*^del^ mice using a Ly6G-specific antibody (anti-mouse GR-1 APC, clone 1A8, 1:200, eBioscience). Cells were incubated with anti-APC beads and separated by MACS cell separation columns following manufacturer’s instruction (Miltenyi Biotec). Neutrophils were labeled with calcein-AM (life technologies) and chemotaxis was measured in response to supernatants of unstimulated or 6 h LPS stimulated BMDMs (5× diluted) in a transwell chemotactic assay over 3-µm pore size fluoroblok filters, as described previously (20).

### Peritoneal Foam Cells

Five mice per group from the atherosclerosis experiment were injected with thioglycollate medium (3%, Fischer). Four days after injection, mice were sacrificed and the peritoneum was flushed once with 10 ml ice-cold PBS to collect peritoneal macrophages (PEMs). Since these mice are on a HFD, these elicited macrophages are fully loaded with lipids and can thus be considered foam cells (21). Flushed thioglycollate-elicited foam cells were pooled per group and cultured at a density of 5 × 10^5^ cells/well in 24-well tissue culture plates in triplicate (Greiner Bio-One) and cultured in RPMI-1640 with 25 mM HEPES, 2 mM L-glutamine, 10% FCS, penicillin (100 U/ml), and streptomycin (100 μg/ml) (all Gibco) and allowed adherence for 3 h. Hereafter the cells were washed and stimulated for 24 h with LPS (10 ng/ml), LPS plus IFNγ (100 U/ml), IL-4 (20 ng/ml), or left unstimulated.

### ELISA and Nitric Oxide Assay

Antibody pair kits were used to measure TNF, IL-6 and IL-12p40 (Life technologies) with ELISA following manufactures instructions. The following sample dilutions were used, TNF 1:10; IL-6 and IL-12p40 1:20. Streptavidin HRP was used to detect bound antibodies and TMB (Thermo scientific) was used as a substrate for the color reaction. This reaction was stopped with 1.8 M H_2_SO_4_. Absorbance was measured on a microplate reader (Victor) at a wavelength of 450 nm. For the nitric oxide (NO) assay, 50 µl of undiluted samples was used on a flat bottom 96-wells plate. A standard curve was made with NaNO_2_ in culture medium. Next 50 µl of Griess reagent (2.5% H_3_PO_4_, 1% Sulfanylamide, 0.1% Napthyleene diamine in MilliQ) was added to the samples and absorbance was immediately measured on the microplate reader at a wavelength of 550 nm.

### Statistical Analysis

Data is presented as mean ± SEM. Statistical analyses were performed using GraphPad Prism 5.0 software using an unpaired *t*-test or two-way ANOVA with bonferroni post-hoc analysis when comparing multiple groups. *P*-values < 0.05 were considered statistically significant.

## Results

### No Differences in the Lipid Levels and Immune Cell Populations After Bone Marrow Transplantation to *Ldlr*^−/−^ Mice

**Figure 1:**
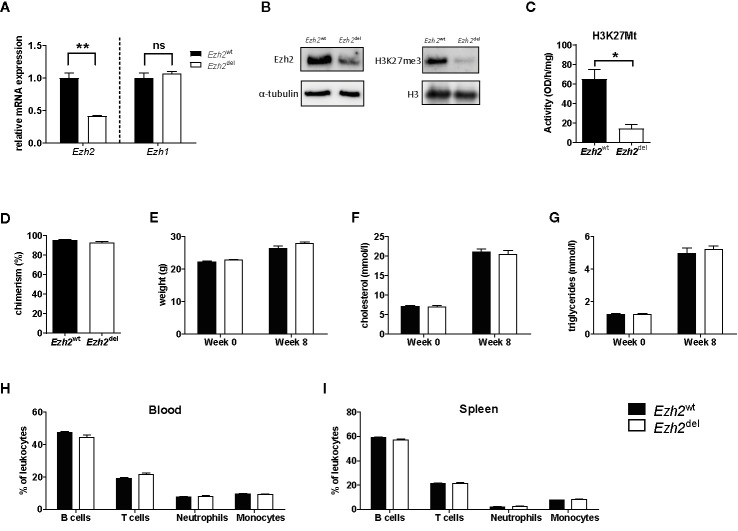
Bone marrow of *Ezh2*^wt^ and *Ezh2*^del^ mice was effectively transplanted to *Ldlr^-/-^* mice. **(A)** Relative normalized *Ezh2* and *Ezh1* mRNA expression in *Ezh2*^wt^ and *Ezh2*^del^ BMDMs **(B)** EZH2 protein expression on whole cell lysates (left) and H3K27Me3 levels on histone extracts (right) of BMDMs. α-tubulin and H3 were used as loading control **(C)** H3K27 methyltransferase activity in nuclear lysates of BMDMs. **(D)** Chimerism determination by qPCR for the *Ldlr* in the DNA of blood of *Ezh2*^wt^ (black bars) and *Ezh2*^del^ (white bars) transplanted mice. **(E)** Mouse weight in grams at the start of the diet (week 0) and after 8 weeks of HFD (week 8). **(F)** Cholesterol and **(G)** triglyceride levels in the plasma at the start and after 8 weeks of HFD. Percentage of **(H)** blood and **(I)** spleen leukocyte subsets assessed by flow cytometry. B cells (CD45^+^, CD19^+^), T cells (CD45^+^, CD3^+^), Neutrophils (CD45^+^, CD11b^+^, Ly6G^+^) and monocytes (CD45^+^, CD11b^+^, Ly6G^-^). N=15 each group. Data represent mean ± SEM.

A myeloid-specific *Ezh2* deficient mouse strain (*Ezh2*^del^) was generated and cultured bone marrow-derived macrophages (BMDMs) showed a reduction of *Ezh2* at both the mRNA as well as protein level, without changes in *Ezh1* mRNA expression (**Figures 1A, B**). EZH1 is most likely not compensating for the loss of EZH2 in macrophages as global H3K27Me3 levels and H3K27 methyl transferase activity was strongly reduced in *Ezh2*-deficient BMDMs (**Figures 1B, C**). Bone marrow of *Ezh2*^wt^ or *Ezh2*^del^ mice was transplanted to *Ldlr*^-/-^ mice, which were subsequently fed a high fat diet (HFD) for 9 weeks to induce atherosclerosis. Bone marrow of both *Ezh2*^wt^ and *Ezh2*^del^ mice was effectively transplanted to *Ldlr*^-/-^ mice as chimerism was around 95% and not different between the groups (**Figure 1D**). Weight, cholesterol and triglyceride levels were similar after 8 weeks of HFD between *Ezh2*^wt^ and *Ezh2*^del^ transplanted mice (**Figures 1E–G**). Additionally, leukocyte levels in both blood and spleen were unaltered in *Ezh2*^del^ transplanted mice compared to wildtype (**Figures 1H, I**). Overall, no differences in the lipid levels or composition of immune cell populations were seen after transplantation of *Ezh2*^del^ bone marrow compared to wildtype, giving similar baseline characteristics.

### Atherosclerotic Lesion Size Is Reduced in *Ezh2*^del^ Transplanted Mice

**Figure 2:**
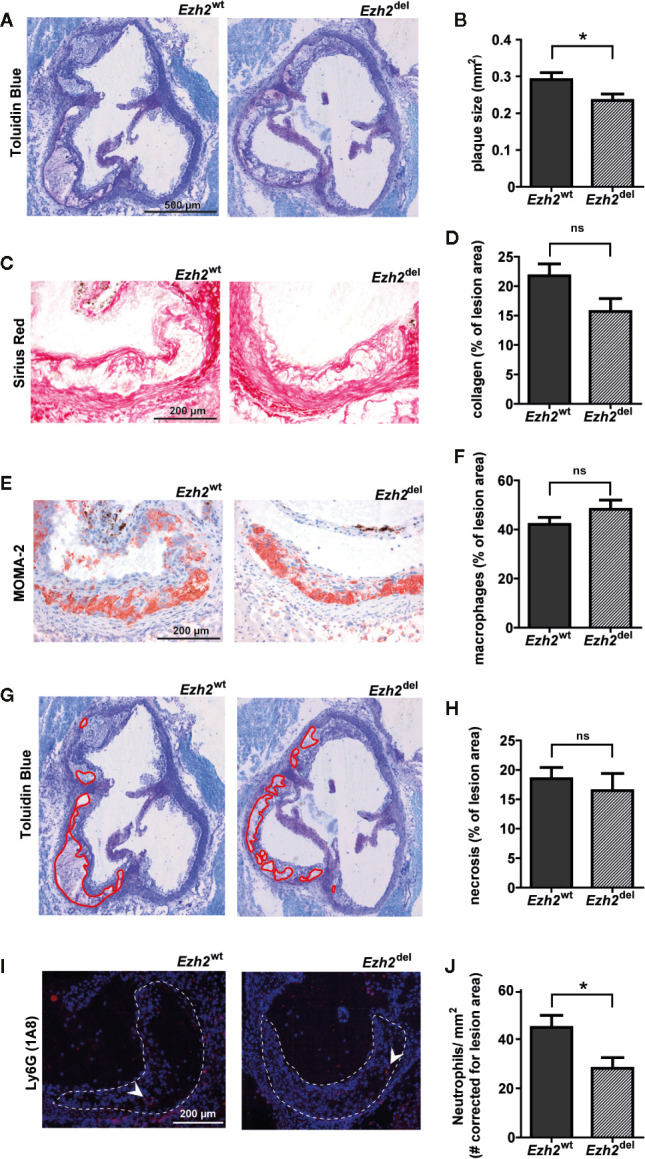
Atherosclerotic lesions size is reduced in *Ezh2*^del^ transplanted mice. **(A)** Representative Toluidin Blue staining of the aortic root of *Ezh2*^wt^ and *Ezh2*^del^ transplanted mice. **(B)** Aortic lesion area presented as the sum of the three valves per mice. **(C)** Representative Sirius Red staining to measure collagen content. **(D)** Collagen content as percentage of total lesion area. **(E)** Representative MOMA-2 staining for macrophages. **(F)** Macrophage area as percentage of total lesion area. **(G)** Necrotic core area indicated in red on the toluidine blue staining. **(H)** Necrotic core as percentage of total lesion area. **(I)** Ly6G (1A8) neutrophil staining in red and nuclei in blue (DAPI). **(J)** Neutrophil counts, corrected for total lesion area. Data represent mean ± SEM of 15 mice. *P < 0.05.

Interestingly, we observed that atherosclerotic lesion size was significantly reduced in *Ezh2*^del^ transplanted mice compared to *Ezh2*^wt^ mice after 9 weeks of HFD (**Figures 2A, B**). A small but non-significant decrease in collagen content was seen in *Ezh2*^del^ transplanted mice (*P* = 0.0528) (**Figures 2C, D**) and the percentage of macrophage and necrosis area were not different between *Ezh2*^wt^ and *Ezh2*^del^ transplanted mice (**Figures 2E–H**). Interestingly, the number of neutrophils was significantly lower in lesions of *Ezh2*^del^ transplanted mice, even when corrected for lesion area (**Figures 2I, J**). These data indicate that myeloid *Ezh2* deficiency leads to atherosclerotic lesions that are not only smaller but also less inflammatory.

### *Ezh2*^del^ Neutrophils Are Less Migratory

**Figure 3:**
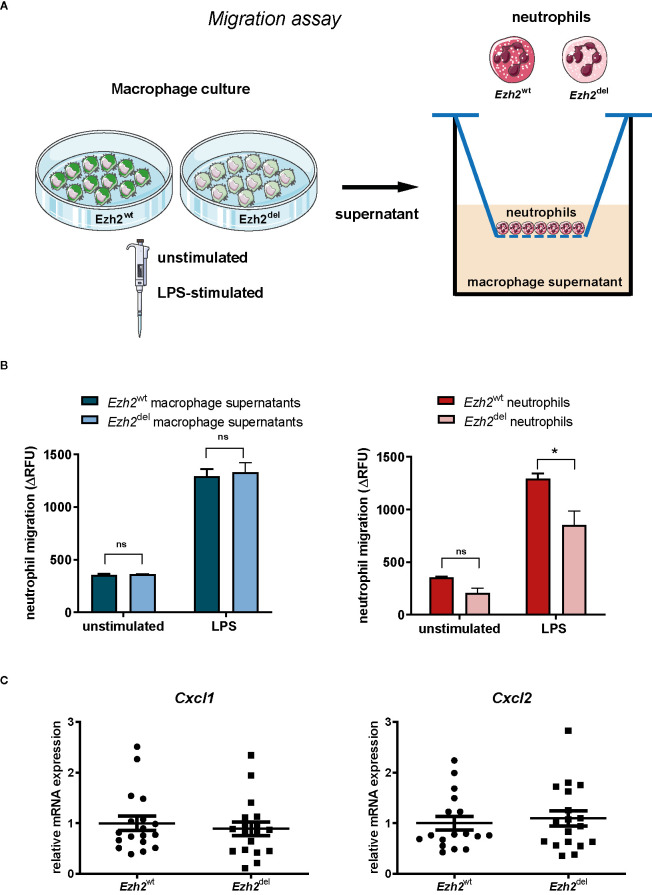
EZH2 regulates neutrophil migration. **(A)** Neutrophils were isolated from bone marrow of *Ezh2*^wt^ or *Ezh2*^del^ mice. Neutrophils were fluorescently labeled and chemotaxis was measured in response to supernatants of unstimulated or LPS activated *Ezh2*^wt^ or *Ezh2*^del^ BMDMs. **(B)** Neutrophil migration as the increase of RFU in time in relative fluorescence units (delta RFU). Presented is one out of three experiment of 2 pooled *Ezh2*^wt^ and 2 pooled *Ezh2*^del^ mice plated in duplo. Left is *Ezh2*^wt^ neutrophil migration toward supernatant of *Ezh2*^wt^ or *Ezh2*^del^ BMDMs and right is *Ezh2*^wt^ or *Ezh2*^del^ neutrophil migration toward wildtype supernantants. **(C)** *Cxcl1* and *Cxcl2* mRNA expression in the aortic arch of *Ezh2*^wt^ or *Ezh2*^del^ transplanted mice. Statistics are performed on duplicates with a two-way anova with bonferroni correction. Data represent mean ± SEM. *P < 0.05. *Panel* **(A)** *was made with use of smart servier medical art, lincesed under a Creative Common Attribution 3.0 Unported License*. https://smart.servier.com/.

Since blood neutrophil numbers were unaltered (**Figure 1E**) but were significantly lower in *Ezh2*^del^ lesions, our data suggest that the migration or recruitment of neutrophils to atherosclerotic lesions is reduced. We next investigated if this was due to differences in the chemotactic factors secreted by *Ezh2*^del^ macrophages or due to intrinsic migration defects of neutrophils. We isolated neutrophils from bone marrow of *Ezh2*^wt^ and *Ezh2*^del^ mice and performed chemotaxis assays. We also assessed wild type neutrophil migration toward supernatants of stimulated *Ezh2*^wt^ and *Ezh2*^del^ BMDMs (**Figure 3A**). We observed no differences in the migration of wild type neutrophils toward supernatants of unstimulated and LPS activated *Ezh2*^wt^ and *Ezh2*^del^ BMDMs (**Figure 3B**, left). Furthermore, the expression of *Cxcl1* and *Cxcl2* expression as the main chemoattractants for neutrophils, was not different in aortic arches of *Ezh2*^wt^ and *Ezh2^d^*^el^ transplanted mice, suggesting that these are similar in the lesion environment and not causing the difference in neutrophil accumulation (**Figure 3C**). Analysis of *Cxcl1* and *Cxcl2* mRNA expression in peritoneal foam cells and BMDMs was inconclusive, as expression was partly increased, which does not support the reduced neutrophil numbers *in vivo* (**Supplemental Figure S1**). Conversely, we did observe that the migration of *Ezh2*^del^ neutrophils itself was reduced compared to *Ezh2*^wt^ neutrophils *in vitro* (**Figure 3B**, right). These data suggest that neutrophils of *Ezh2*^del^ mice are less migratory and therefore accumulate less in the atherosclerotic lesions.

### Inflammatory Responses Are Partly Reduced in *Ezh2*^del^ Activated Peritoneal Foam Cells

**Figure 4:**
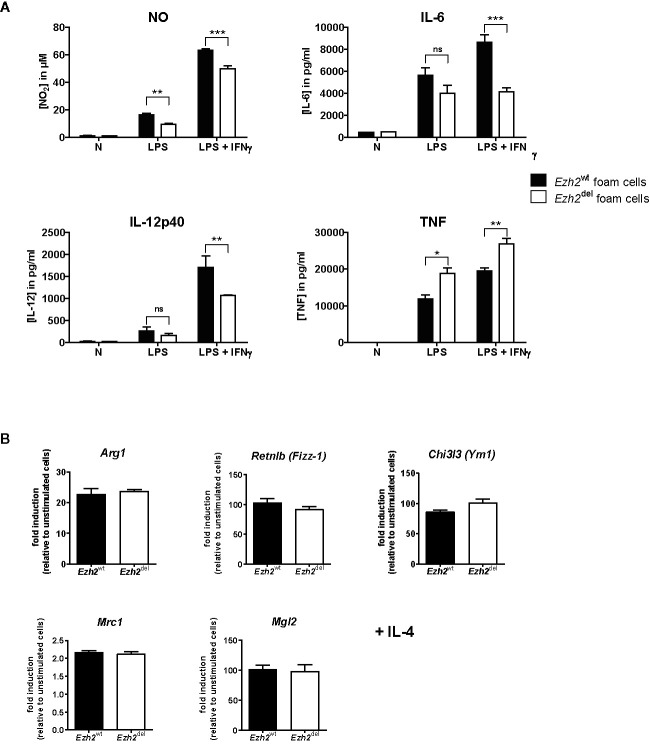
EZH2 affects inflammatory responses in peritoneal foam cells. **(A)** Cytokine production by *Ezh2*^wt^ (black bars) and *Ezh2*^del^ peritoneal foam cells after stimulation for 24 h with LPS or LPS+IFNγ. **(B)** mRNA expression in *Ezh2*^wt^ and *Ezh2*^del^ peritoneal foam cells after 24 h of IL-4 stimulation. Foam cells of five individual mice per group were pooled and plated in triplicate. Data represent mean ± SEM. *P < 0.05; **P < 0.01; ***P < 0.001.

Since Zhang et al. showed that *Ezh2* deficiency diminishes the pro-inflammatory response of microglia and macrophages, we here studied whether this is also true for macrophages under HFD conditions, i.e. foam cells. Thioglycollate elicited peritoneal macrophages were isolated from transplanted HFD-fed mice and studied *ex vivo*. Since these mice are on a HFD, these macrophages are fully loaded with lipids and can thus be considered foam cells (21). Peritoneal foam cells from *Ezh2*^del^ mice produced less NO, IL-6, and IL-12p40 after 24 h of LPS+IFNγ stimulation compared to *Ezh2*^wt^ foam cells, indicating that the inflammatory response is reduced in *Ezh2*^del^ foam cells (**Figure 4A**). However, TNF secretion was enhanced in activated *Ezh2*^del^ foam cells (**Figure 4A**). We also activated these foam cells with IL-4 as an inducer of alternative macrophage activation but did not observe any changes in IL-4 responsive gene expression (**Figure 4B**). The inflammatory response is thus partly reduced in *Ezh2*^del^ foam cells without changes in IL-4 responsive genes.

## Discussion

We here studied the role of the H3K27 methyltransferase EZH2 in atherosclerosis development by use of a myeloid-specific *Ezh2* knockout mice. *Ezh2*-deficient mice showed reduced levels of H3K27me3 and lower H3K27 methyltransferase activity, indicating that EZH1 is not compensation for the loss of EZH2. *Ezh2* mRNA levels were partly reduced (± 60%) as deletion is never complete in the used floxed LysM-cre system and recombination efficiency dependent on the genomic location and distance between the LoxP sites (22–24). As our LoxP sites flank the catalytic SET domain of *Ezh2*, it is sufficient to strongly reduce H3K27Me3 levels and H3K27 methyltransferase activity.

We show that myeloid *Ezh2* deficiency improves atherosclerosis outcome as lesion size was significantly reduced in *Ezh2*^del^ transplanted mice compared to wildtype. Overexpression of *Ezh2* in all cell types was previously reported to promote macrophage foam cells formation and worsen atherosclerosis outcome (25). In line with their findings, we show that silencing of *Ezh2* specifically in myeloid cells reduces atherosclerosis. Moreover, we show that atherosclerotic lesions of myeloid *Ezh2*-deficient mice are not only smaller but also contain less neutrophils. Neutrophils play an important role in all stages of atherosclerosis (26) and neutrophil depletion studies in atherosclerotic mice (*ApoE*^-/-^) showed a reduction in early atherosclerosis (27). Since neutrophil numbers were similar in blood, this implies that the recruitment of neutrophils toward the lesion is impaired.

We studied neutrophil migration *in vitro* and indeed found that *Ezh2*-deficient neutrophils are less migratory. This phenomenon is consistent with studies showing that neutrophils derived from *Ezh2* knockout stem cells have impaired migration together with increased cell death, decreased phagocytosis and overproduction of reactive oxygen species (ROS) (28). Accordingly, Gunawan et al. observed reduced neutrophil migration and in addition found reduced dendritic cell migration in cells lacking *Ezh2* (29). They propose a mechanism by which EZH2 regulates migration, independent of its H3K27 methyltransferase activity (29). They show that EZH2 regulates integrin mediated migration *via* methylation of Talin1. EZH2 interacts with VAV1, causing methylation of Talin1. In turn, binding to filamentous actin (F-actin) was altered affecting adhesion and migration.

Likewise, we show that the inflammatory response of peritoneal foam cells is reduced in activated *Ezh2*^del^ cells compared to *Ezh2*^wt^ cells. IL-6, IL-12 and NO production, which are key cytokines induced upon macrophage activation, were reduced in *Ezh2*^del^ foam cells. Interestingly, the inflammatory cytokine TNF was increased in cells lacking *Ezh2*, indicating that not the full activation spectrum is impaired. Additionally, IL-4 responsive genes were not affected. The idea that inhibition of EZH2, a mediator of repression, results in enhancement of the inflammatory gene program in macrophages appears to be too simplistic. Zhang and colleagues nicely demonstrated one of the mechanisms by which the repressive EZH2 causes activation of inflammation in macrophages (10). By chromatin immunoprecipitation (ChIP) and RNA sequencing analysis they identified the suppressor of cytokine signaling 3 (Socs3) as being strongly upregulated in the absence of EZH2 in combination with EZH2 binding sites and reduced H3K27me3 levels in macrophages. They show that *Ezh2* deficiency lowers Socs3 levels thereby enhancing Traf6 ubiquitination and degradation. The Myd88-NF-κB signaling pathway is thereby suppressed, resulting in the reduced inflammatory responses. They illustrate how a repressor like EZH2 can cause activation of inflammation by repression of another repressor, in this case SOCS3 (9, 10). Additionally, *Ezh2* deficiency improved the outcome on EAE and colitis, two inflammatory mouse models. We here show that myeloid *Ezh2* deficiency also improves the outcome of atherosclerosis, another chronic inflammatory disease.

In conclusion, we show that myeloid *Ezh2* deficiency limits atherosclerosis due to impaired neutrophil migration and modulation of foam cell inflammatory responses. Our findings support the thought that EZH2 is not only a target for various types of cancer but may also serve as novel target for auto-immune diseases. Several EZH2 inhibitors are available and the use of these inhibitors in mouse models of atherosclerosis would be the next step to test the therapeutic potential of EZH2 inhibition to control atherosclerosis.

## Data Availability Statement

The raw data supporting the conclusions of this article will be made available by the authors, without undue reservation.

## Ethics Statement

The animal study was reviewed and approved by the Committee for Animal Welfare of the Amsterdam UMC, location Academic Medical Center, University of Amsterdam (permit: DBC10AH).

## Author Contributions

AN and MW conceived and designed the experiments. AN, H-JC, MG, SV, MH, MB, JB, AT, and HM performed the experiments. AN, MG, and SV analyzed the data. AN, MG, EL, and MW were involved in the interpretation of the data. AN and MW wrote the manuscript. MG, JB, TB, and EL critically read and revised the manuscript. All authors contributed to the article and approved the submitted version.

## Funding

This work was supported by The Netherlands Heart Foundation [CVON 2011/B019 and CVON 2017-20: Generating the best evidence-based pharmaceutical targets for atherosclerosis (GENIUS I&II)]. AN received a Junior Postdoc grant from the Netherlands Heart Foundation (2020T029) and a postdoc grant from Amsterdam Cardiovascular Sciences. JB received a senior postdoctoral fellowship (2017T048) from the Netherlands Heart Foundation. MW is an established investigator of the Netherlands Heart Foundation (2007T067), is supported by a Netherlands Heart Foundation grant (2010B022), The Netherlands Heart Foundation and Spark-Holding BV (2015B002, 2019B016), the European Union (ITN-grant EPIMAC), Leducq Transatlantic Network Grant, ZonMW (Open competition 09120011910025) and holds an AMC-fellowship. MW and EL are both supported by REPROGRAM (EU Horizon 2020).

## Conflict of Interest

The authors declare that the research was conducted in the absence of any commercial or financial relationships that could be construed as a potential conflict of interest.
