# Supplementary material for: Myeloid Ezh2 Deficiency Limits Atherosclerosis Development
Source: Front Immunol. 2021 Jan 26;11:594603. doi: 10.3389/fimmu.2020.594603 (PMC7871783; doi:10.3389/fimmu.2020.594603)
Supplement: Supplementary file 2 [file Table_1.docx]

**Supplemental Material**

**Supplemental Table 1: Antibody specifications**

| Antibody | Dilution | Supplier | Clone | Order# |
| --- | --- | --- | --- | --- |
| **CD45 (APC-Cy7)** | 1:100 | Biolegend | 30-F11 | 103116 |
| **CD11b (FITC)** | 1:100 | eBioscience | M1/70 | 11-0112 |
| **Ly6G (PE)** | 1:200 | Biolegend | 1A8 | 127608 |
| **CD19 (PerCp Cy5.5)** | 1:100 | eBioscience | 1D3 | 45-0193 |
| **CD3 (FITC)** | 1:100 | eBioscience | 145-2C11 | 11-0031 |

**Supplemental Figure 1: Expression of neutrophil chemoattractant genes (A)** Relative normalized *Cxl1* and *Cxcl2* mRNA expression in *Ezh2^wt^* and *Ezh2*^del^ peritoneal foam cells after 3h LPS stimulation **(B)**  Relative normalized *Cxl1* and *Cxcl2* mRNA expression in *Ezh2^wt^* and *Ezh2*^del^ BDMS after 3h LPS stimulation. Data represent mean ± SEM.
